# Current State of Cartilage Tissue Engineering using Nanofibrous Scaffolds and Stem Cells

**Published:** 2017

**Authors:** Somaieh Kazemnejad, Manijeh Khanmohammadi, Nafiseh Baheiraei, Shaghayegh Arasteh

**Affiliations:** 1. Reproductive Biotechnology Research Center, Avicenna Research Institute, ACECR, Tehran, Iran; 2. Department of Anatomical Sciences, Faculty of Medical Sciences, Tarbiat Modares University, Tehran, Iran

**Keywords:** Cartilage, Nanofibers, Scaffolds, Stem cells, Tissue engineering

## Abstract

Cartilage is an avascular, aneural, and alymphatic connective tissue with a limited capacity caused by low mitotic activity of its resident cells, chondrocytes. Natural repair of full thickness cartilage defects usually leads to the formation of fibrocartilage with lower function and mechanical force compared with the original hyaline cartilage and further deterioration can occur. Tissue engineering and regenerative medicine is a promising strategy to repair bone and articular cartilage defects and rehabilitate joint functions by focusing on the optimal combination of cells, material scaffolds, and signaling molecules. The unique physical and topographical properties of nanofibrous structures allow them to mimic the extracellular matrix of native cartilage, making an appropriate resemblance to induce cartilage tissue regeneration and reconstruction. To improve simulation of native cartilage, the incorporation of nanofibrous scaffolds with suitable corresponsive cells could be effective. In this review article, an attempt was made to present the current state of cartilage tissue engineering using nanofibrous scaffolds and stem cells as high proliferative immune privilege cells with chondrogenic differentiation ability. The comprehensive information was retrieved by search of relevant subject headings in Medline/Pubmed and Elsevier databases.

## Introduction

Because of limited capacity for spontaneous repair, cartilage tissue cannot be restored to its normal function and structure after damages caused by trauma, osteoarthritis disease, accidents and so forth. Surgical strategies to repair cartilage chondral or osteochondral defects have been used to restore joint function and eliminate associated pain, including stimulation of the marrow by microfracture, mosaicplasty and cell-based therapies. Although surgical strategies reduce patient pain and increase joint mobility, the regenerated tissue is morphologically, biochemically and biomechanically inferior to the native cartilage. Additional surgery is often required to regain complete function, resulting in the progression to partial or total knee replacement. Therefore, there is a tremendous need for new regenerative medicine approaches to augment the repair process and to facilitate adequate tissue regeneration and longevity.

The novel strategy for regeneration of cartilage defects involves cells seeded biomaterials with appropriate growth factors ^1^. Biomaterial as a proper microenvironment for the cells provides mechanical support for engineered tissues. Recently, commercially available synthetic and natural matrix has been tested in animal models or clinical trials for repair of cartilage and the overall short-term clinical outcome is favorable ^2^. Therefore, tissue engineering is a promising option for the treatment of cartilage defects. Using different materials and production methods, many forms of biomaterial scaffolds with different properties have been developed for cartilage tissue engineering.

In the past decade, nanofibrous structures have attracted much interest as tissue engineered scaffolds because of their unique physical and topographical properties. The nanosized structure of a scaffold plays an important role to mimic the Extracellular Matrix (ECM) Structure ^3^.

Nanofiber scaffolds composed of ultra-fine biodegradable polymeric fibers morphologically similar to natural ECM have been widely emerged as potential scaffolds for cartilage tissue engineering.

It is worth mentioning that while nanofibrous structures could mimic similar fiber diameters, composition, and alignment of the ECM of articular cartilage, the synchronization of these scaffolds with suitable corresponsive cells could help us to achieve the best tissue engineering results for articular cartilage ^4^. Due to some characteristics of stem cells such as self-renewal, high proliferation and trans-differentiation capacity that reduce the challenges propounded about chondrocytes ^5^, these non-specialized cells are the focus of interest in tissue engineering and regenerative medicine field. This article reviewed and presented actual status of *in vitro* and *in vivo* studies on the application of nanofibrous structures and stem cells for cartilage tissue reconstruction. For extraction of related publications, keywords of cartilage tissue engineering, nanofibers and stem cells as MeSH terms in PubMed were used. All data belong to the publications and efforts in the field of cartilage tissue engineering and nanofibers that was achieved to date.

## Different methods for fabrication of nanofiber scaffolds

Different synthetic nanomaterials have been fabricated to create the microenvironment that seeded cells can be encouraged to expand and differentiate into desired lineages, including chondrocytes ^6,7^. The biomemetic properties and good physiochemical features of nano-materials play a key role in stimulation of chondrocyte growth and cartilage tissue regeneration ^8,9^. Their physical characteristics promote advantageous biological responses of seeded cells *in vitro*, including increased cell proliferation and attachment while maintaining chondrocytic phenotype ^9,10^. In addition, application of nanofibrous scaffolds enables incorporation of nanospheres containing different growth factors. Exogenous transforming growth factor (TGF-β) family has been proved to stimulate cell proliferation and chondrogenesis both *in vivo* and *in vitro*. The factor TGF-β1 is naturally found in human platelets, bone, and other tissues and has been shown as an inducer of chondrogenesis ^11^. The controlled release of Bone Morphogenetic Protein (BMP-7) from nanospheres-containing scaffolds has induced significant ectopic bone formation *in vivo* ^12^. Based on these findings, nanofibrous scaffold and nanospheres, combined with chondrogenic and osteogenic factors, have been introduced as potential candidates to reconstruct the osteochondral defect for the regeneration of bone, cartilage, and their interface simultaneously ^13^. To provide ECM-like nanofibrous scaffolds, a variety of techniques have been developed, including electrospinning, self-assembly, phase separation and drawing ^14^.

### Electrospinning

The most conventional method for processing of polymeric biomaterials into nanofibrous scaffolds is electrospinning with promising results for tissue engineering applications. This process is a simple economical technique to produce nanofibers from a wide range of synthetic and natural polymers in randomly-oriented or aligned manner ^10^. Electrospun nanofibers have a high specific surface area and can be functionalized with bioactive macromolecules ^15,16^. Electrospinning outcome is influenced by several parameters, including molecular weight of polymer, polymer solution properties, electric potential, distance between capillary and metal collector, *etc*.

In spite of the benefits electrospinning has to offer, it suffers from limitations including jet instability, toxic solvent, packaging, handling ^17^, and the production of two-dimensional (2D) matrices with small pores, which inhibits cell penetration and vascular ingrowth ^18^. In order to elicit the maximum benefit from this method, there are some advancements or modifications to the processing conditions ^19^. Coaxial electrospinning technique ^20^ enables the controlled release of active bio-molecules by producing core-shell nanofibers trapping drugs or bioactive molecules. Several attempts have been made to fabricate three-dimensional macroporous nanofibrous electrospun scaffolds by modifying the electrospinning conditions or using post-treatments. Process modifications include low-temperature electrospinning ^21^, needleless electrospinning using disc as spinneret ^22^, application of different collector plates, such as parallel plate ^23^ and screws ^24^, and introducing micrometer-sized fibers ^25–27^ or inert particle spacers, such as salts ^28,29^, poly(ethylene oxide) (PEO) ^30^, gas ^31^, *etc*. Using solutions with polyelectrolyte nature (a high charge density material) leads to the extension of fibers outwards from the collector under conditions which induce repulsion between neighboring fibers ^32^. In brief, post-treatments include photo-masking ^33^ or stacking layered mats ^34^.

### Self-assembly

Novel nanofibrous scaffolds have been fabricated by self-assembling peptides through molecular self-assembly by mimicking regulatory mechanisms of natural ECM. Self-assembly is a manufacturing process in which small molecules-as basic building blocks- will be added-up to form nanofibres. These structures have gained much progress in repairing different injured tissues such as cartilage, bone, nerve, heart and blood vessel ^35^. Two significant approaches have been proposed to proximate peptide nanofiber scaffolds to ECM: (1) modification with functional motifs (*e.g*. RGD, IKVAV and YIGSR) and (2) controlled release of molecular signals such as Fibroblast Growth Factor (FGF-2) and Vascular Endothelial Growth Factor (VEGF). In self-assembly, intermolecular forces determine the properties and shape of nanofibers. Nanofibers can be assembled with various polymeric configurations such as diblock copolymers, triblock copolymers, triblock polymers (of peptide amphiphile and dendrimers), and bolaform (of glucosamide and its deacetylated derivatives) ^36^. *In vitro* assessment of many peptide nanofiber scaffolds have revealed the ability to induce cell proliferation, differentiation, migration and ECM production ^37–39^. Poor mechanical property of peptide nanofiber scaffolds might limit its application to non-load-bearing sites ^40^.

### Phase-separation

Phase-separation is a method for fabrication of 3D nanofibrous structures with nanofibers that closely mimic dimension of collagen fibrils of ECM (50–500 *nm*) ^41^. This technique is based on the physical incompatibility of polymers and their tendency to separate into two phases for nanofiber production ^17^. Phase-separation provides the possibilities of scaffold fabrication for a desired anatomical shape and presenting the nano and macro architecture simultaneously ^42^. Although the fabrication process is convenient and requires simple instrumentation, it is limited to only certain specific polymer-solvent combinations. Also, fiber dimensions cannot be controlled and the mechanical properties of the fiber are not suitable for load-bearing applications due to the highly porous structure. The controlling parameters include polymer type, polymer concentration, solvent type and thermal treatment ^41^.

### Drawing

In the drawing process, a micropipette, a few micrometers in diameter, is dipped into a polymer liquid and withdrawn at a fixed speed resulting in production of nanofibers. This process is simple and is suitable for viscoelastic materials bearing strong deformations while being united enough to support the stresses developed under pulling. However, it is limited to laboratory scale as nanofibers are formed one by one. Another limitation is that, there is no control on fiber dimensions and only fibers with diameters in the micrometer size can be produced. Also, an additional step such as weaving is needed to make scaffolds for tissue engineering applications ^17,36^.

Some advantages and disadvantages of the above mentioned techniques in terms of their fabrication, reproducibility and controllability have been summarized in table 1.

**Table 1.** Advantages and disadvantages of different methods for fabrication of nanofibers

**Manufacturing process**	**Control on fiber dimension**	**Advantages**	**Disadvantages**
**Electrospinning**	Yes (from few nanometers to several microns)	- Continuous process - Cost effective - Simple instrument - Producing both random and oriented nanofibers - High porosity and surface area	- Fiber thickness - No control over 3D pore structure - Jet instability
**Drawing**	No	- Simple process - Simple equipment	- Discontinuous process - Time consuming - Applicable only to viscoelastic materials - Low productivity
**Phase-separation**	No	- Simple equipment - Simple procedure - Tailorable mechanical prop	- Only works with limited number of polymers - No control on fiber alignment - Low productivity
**Self-assembly**	No	- Easy to get smaller nanofibres - Structure varieties (layered and lamellar)	- Complex procedure - Low productivity - No control on fiber alignment - Limitation on polymers

### The advantages of stem cells for cartilage tissue engineering purposes

In native tissues, cells are constantly interacting with the surrounding ECM that leads to transferring information between the extracellular and intracellular space, directing their behavior. Chondrocytes are the sole cell type in articular cartilage that mostly has been served as the cell source for articular cartilage repair in clinic. However, their utilization in clinic is accompanied with some limitations. For example, autologous chondrocyte availability is limited and cannot provide the high cellular demand of articular cartilage repair. Although some *in vitro* cell expansion methods have been developed to increase cell numbers for transplantation, the risk of chondrocytes dedifferentiation during *in vitro* culture is a big challenge ^43,44^.

Although there exists a wide range of studies on transplantation of more available chondrocyte sources such as allogeneic or xenogeneic chondrocytes instead of autologous chondrocytes, these chondrocytes can potentially induce immune responses or transmit diseases. Thus, the application of allogeneic and xenogeneic chondrocytes requires further investigations to remove such concerns. Since chondrocytes from each of the four zones exhibit different properties, another strategy is the use of separately seeded zonal chondrocytes toward regenerating biomimetic functional cartilage tissue ^45,46^. Due to the aforementioned limitations of chondrocyte sources, there is much effort to find out alternative cell sources. In these years, fascinating characteristics of stem cells especially adult stem cells such as accessibility, availability and chondrogenic capacity have introduced these cells as promising cell sources for articular cartilage tissue engineering ^5^.

Embryonic Stem Cells (ESCs) and induced Pluripotent Stem Cells (iPSCs) are cell sources with high chondrogenic potentials; however, there are concerns on their immunogenicity, potential for malignancy, ethical issues (for ESCs), and heterogeneous differentiation. Therefore, these cell sources cannot be the best candidate for cartilage tissue engineering ^47^.

**Figure 1.**
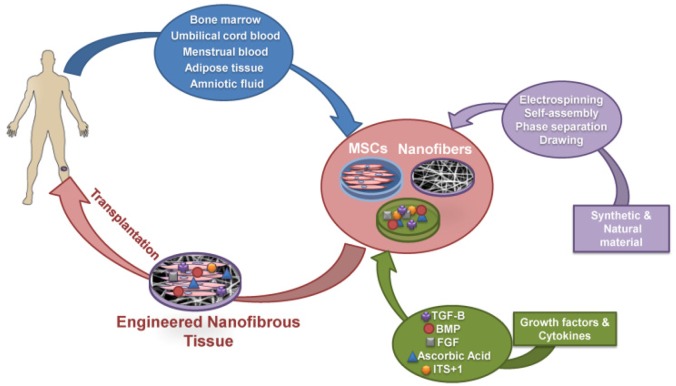
Schematic diagram of cartilage tissue engineering process using nanofibers and stem cells. Mesenchymal stem cells derived from different sources are expanded *ex vivo* and subsequently cultured in nanofiber scaffolds to initiate differentiation in presence of growth factors and cytokines. Finally, the engineered nanofibrous tissues were implanted *in vivo* for cartilage tissue regeneration. MSCs: Mesenchymal Stem Cell, BMP: Bone Morphogenetic Protein, TGF-B: Transforming Growth Factor-Beta, FGF: Fibroblast Growth Factor, ITS+1: Insulin-Transferrin-Selenium+ Bovine Serum Albu*min* and Linoleic Acid.

As shown in figure 1, adult stem cells being derived from different tissues such as bone marrow, cord blood, placenta, adipose tissue, amniotic fluid and menstrual blood combined with nanofibrous scaffolds have been widely used for cartilage tissue engineering ^13,48–50^. Compared with adult chondrocytes, they can easily be obtained and manipulated as they are able to undergo several passages before losing their differentiation potential.

Bone Marrow Mesenchymal Stem Cells (BMMSCs), for example, are multi-potential stem cells with the capacity to differentiate into a variety of tissue types including bone, cartilage, fat, muscle, tendon and other tissues when induced by the appropriate cues both *in vitro* or *in vivo* ^51,52^. They can provide a suitable cell source for osteochondral tissue reconstruction ^13^ and have also been previously explored for the engineering of fibro-cartilaginous tissues such as the annulus fibrous of the inter-vertebral disc and the knee meniscus ^53,54^. For example, Li *et al* stated that adult BMMSCs seeded on electrospun polycaprolactone (PCL) combined with TGF-β1 differentiated into a chondrocytic phenotype at levels comparable to traditional pellet cultures. The designed constructs showed a zonal morphology with a layer of cartilaginous matrix composed of collagen type II, cartilage proteoglycan link protein, and aggrecan ^55^.

Shafiee *et al* have studied the *in vitro* characteristics and chondrogenic capacity of four available human adult stem/progenitor cell sources using aligned electrospun polycaprolactone/poly (L-lactic acid) (PCL/PLLA) nanofibers. The studied cells include BMMSCs, adipose tissue-derived MSC (AD-MSC), Articular Chondrocyte Progenitors (ACP), and nasal septum-derived progenitors (NSPs). Accordingly, NSPs exhibited the highest proliferation potential and chondrogenic capacity ^47^.

More recently, menstrual blood has been identified as an easily accessible and renewable stem cell source with the higher proliferative rate compared with umbilical cord and bone marrow derived mesenchymal stem cells ^56,57^. Our group presented the evidence introducing menstrual blood stem cells (MenSCs) as a suitable stem cell population candidate for cartilage tissue engineering. Indeed, the chondrogenic capacity of MenSCs is a major issue which may support future application of MenSCs as a reliable source for cell therapy of cartilage defects.

## *In vitro* findings on recapitulation of ECM environment for cartilage tissue engineering using nanofibrous scaffolds

### Single polymer-based nanofibrous matrices

Electro-spun nanofibers with different compositions have been widely studied for osteochondral differentiation (Table 2). Chondrogenic differentiation of BMMSCs has been extensively studied on 2D electrospun nanofibrous matrices using single polymer, such as PCL ^58,59^ and poly (D,L-lactide-co-glycolide) (PLGA) ^60,61^. Wise *et al* found that cell orientation is minimally influenced by soluble factors and is mainly controlled by physical cues (oriented micro- and nano-fibers in this study); however, cell shape was affected by chondrogenic factors ^58^. Cells cultured in chondrogenic media on nanofibers showed a significant increase in the sGAG content and expression of collagen type II in comparison with culturing in normal growth media and on microfiber scaffolds ^58^. Alves da Silva *et al* cultured BMMSCs on electrospun PCL nanofiber mesh in a multi-chamber flow perfusion bioreactor to produce cartilagineous extracellular matrix ^59^. Statically cultured cells had a fibroblast-like morphology, while dynamic condition induced round-shaped morphology with increased amount of sGAG and collagen type I and II. However, there was no significant difference between gene expression of chondrogenic markers in two culture conditions. Another study has shown that PLGA electrospun nanofibers assisted the growth and differentiation of human BMMSCs as well as their osteogenic and chondrogenic potential ^60^.

**Table 2.** *In vitro* studies on cartilage tissue engineering using stem cells and nanofibers

**Species**	**Cells Source**	**Cells Type**	**Biomaterials**	**Stimulating Factors**	**Results**	**Ref.**
**Rabbit**	Bone Marrow	Mesenchymal Stem Cells (MSCs)	Poly (Vinyl Alcohol)/Poly (E-Caprolactone): PVA/PCL	TGF-B1, FGF-2, Dexamethasone, Ascorbate 2-Phosphate, ITS+1 premix,	MSCs seeded on PVA/PCL scaffolds showed the mRNA expression of collagen type II and Aggrecan after 21 days of chondrogenic differentiation	(3)
**Goat**	Bone Marrow	MSCs	Poly (Vinyl Alcohol) - methacrylate (PVA-MA) PVA-hondroitin Sulfate- methacrylate (PVA-CS-MA)	TGF-B1, Ascorbate 2-Phosphate, Dexamethasone, L-Proline, Sodium Pyruvate, ITS-Plus Premix	A higher collagen type II/type I gene expression ratio in PVA-CS-MA compared with PVA-MA fibers alone	(7)
**Fetal Bovine**	Epiphyseal Cartilage	Chondrocytes	PCL	Ascorbate 2-Phosphate, Dexamethasone, Sodium Pyruvate, Proline, ITS-Plus Premix	Chondrocytes seeded on the PCL scaffold maintained their chondrocytic phenotype by gene expressing of collagen types IIB and IX, aggrecan, and cartilage oligomeric matrix protein	(9)
**Human**	Bone Marrow	MSCs	PCL and sodium hyaluronate (HA)	TGF- B1, Bovine Serum Albumin (BSA)	Initial release of HA is sufficient in terms of directing the implanted MSCs toward a chondrogenic end, whereas a late release of TGF-B1 is preferred to foster type II and avoid type I collagen expression	(11)
**Human**	Bone Marrow	MSCs	Poly (L-lactic) acid (PLLA)	TGF- B1	In the presence of TGF- B1, cartilage tissue developed on PLLA scaffolds had high level of Sulfated glycosaminoglycans (sGAG), Sox-9 and collagen type II	(13)
**Human**	Umbilical Cords	MSCs	Poly L-lactide-co-glycolic acid (PLGA) and PCL	TGF-B3, TGF-B1, IGF, BMP6, Ascorbate 2-Phosphate, ITS-Plus Premix, Dexamethasone, L-Proline	Level of sGAG and sulfated proteoglycans and also the ratio of collagen type II to collagen type I expression was up-regulated in differentiated MSCs on PLGA. Cells differentiated on the scaffold	(48)
**Human**	Menstrual blood	Menstrual blood-derived stem cells (MenSCs)	PCL	TGF-B3, IGF-1, Sodium Pyruvate, Ascorbate 2-Phosphate Dexamethasone, ITS+1 premix	had high level of collagen type II and also proteoglycan production compared to 2D system	(56)
**Human**	Bone Marrow	MSCs	PCL	TGF-B1, Ascorbate 2-Phosphate, Sodium Pyruvate, L-Proline, ITS-Plus Premix	Gene expression of collagen types II and IX and also the level of sGAG was up-regulated in nanofibrous system compared with control culture	(55)
**Human**	Bone Marrow	MSCs	PLGA	TGF-B3	MSCs seeded in PLGA nanofiber scaffold in chondrogenic induced medium began to produce high level of sGAG compared to MSCs seeded in PLGA nanofibers without chondrogenic differentiations	(60)
**Bovine**	Carpometacarpal joints of the forelimbs	Chondrocytes	PLLA	TGF-B1, IGF-1, Ascorbate 2-Phosphate, Dexamethasone, Sodium Pyruvate, Proline ITS-Plus Premix	The dynamic culture condition and IGF-1/TGF-b1 treatments upregulated collagen and sGAG production in packed cell nanofiber composite cultures	(88)
**Human**	Placentas	MSCs	nano-sized calcium-deficient hydroxyapatite (nCDHA) and/or a recombinant protein containing arginine–glycine–aspartate (RGD) into the alginate gel and PLGA	TGF-B3, Ascorbate 2-Phosphate, Dexamethasone, l-proline	The amount of sGAG and collagen type II accumulated was found to be the greatest for human Placenta-derived MSCs embedded in the alginate/nCDHA/RGD gel and injected and cultivated in the PLGA scaffold	(50)
	Bone marrow					
**Rat**	Subcutaneous Fat					
**Human**	Bone Marrow	MSCs	PCL	TGF- B1, Ascorbate 2-Phosphate, Sodium Pyruvate, Dexamethasone, l-proline	The expression of collagen type II and aggrecan was upregulated significantly in MSCs seeded on the nanofibrous PCL scaffold	(58)
**Human**	Cartilage	Chondrocytes	Polylactic acid (PLA) microfibers and PCL nanofibers	TGF-B1, Ascorbate 2-Phosphate, ITS+1 premix, Dexamethasone	The pore sizes in the scaffolds were tailored and increased from nanometer scale in purely nanofibrous scaffolds to hundreds of micrometers in scaffolds of nanofiber-coated microfibers. Also, SEM analysis indicated that the chondrocytes adhered and spread on composite scaffolds and produced high level of extracellular matrix.	(89)
**Porcine**	Articular Cartilage	Chondrocytes	PLGA nanofiber and membrane scaffold	Ascorbate 2-Phosphate	The DNA content and normalized sGAG content of the nanofiber based scaffolds were significantly higher than those of the membrane-type scaffolds.	(90)
**Rabbit**	Bone Marrow	MSCs	Natural Nanofibrous Articular Cartilage extracellular matrix (ACECM) and PLGA composite oriented scaffold	-	Cell proliferation test showed that the number of MSCs in ACECM and composite scaffolds was noticeably higher than that in PLGA scaffold, which was coincident with results of SEM observation and cell viability staining	(91)
**Human**	Bone Marrow	MSCs	PCL Microfibers and Nanofibers	TGF-B3, Ascorbate 2-Phosphate, L-proline, Dexamethasone, ITS+1 premix	Cellular proliferation and sGAG and collagen production were enhanced on microfiber in comparison to nanofiber scaffolds, with high initial seeding densities being required for significantchondrogenic differentiation and extracellular matrix (ECM) deposition. Moreover, the collagen type II/I ratio, as a indicator of hyaline cartilage phenotype, was significantly greater for the higher seeding densities on microfibers than nanofibers and in comparison to the lower seeding densities	(92)
**Human**	Adipose Tissue	Adipose-Derived Stem Cells (ASCs)	PCL and cartilage-derived matrix (CDM)	TGF-B1, BMP-6, Dexamethasone,, Ascorbate 2-Phosphate, L-proline	Incorporation of CDM into seeded scaffolds with hASCs stimulated sGAG synthesis and collagen type 10A1 gene expression. Also, compared with single-layer scaffolds, multilayer scaffolds enhanced cell infiltration and ACAN gene expression	(93)
**Human**	UmbilicalCord	Umbilical Cord Wharton’s Jelly Stem Cells (WJSCs)	PCL/Collagen	TGF-B3, FGF-2,L-proline, ITS+1 Premix, Dexamethasone,Ascorbate 2-Phosphate, Sodium Pyruvate	Seeded scaffolds with WJSCs and MSCs showed positive staining in 21 days for the chondrogen related proteins collagen type II and SOX9 and also sGAG values compared to controls	(94)
	Bone Marrow	MSCs				
**Rat**	Bone Marrow	MSCs	PCL nanofibers encapsulated with Hyaluronic acid (HYA) and CS	-	Collagen type II was expressed more in the scaffolds with nanofibers inclusive of CS and HYA than in the scaffolds with vertically oriented nanofibers	(95)
**Human**	Bone Marrow	MSCs	PLLA Microfibers and Nanofibers	TGF-B3, ITS +1premix Dexamethasone, Ascorbic acid-2-phosphate, Sodium Pyruvate, L-proline	Chondrogenic markers of aggrecan, chondroadherin, sox9, and collagen type II were the highest for cells on micron-sized fibers in comparison to cells on nano-sized fibers	(96)
-	-	C3H10T1/2 murine embryonic mesenchymal progenitor cells	core-shell poly(ether sulfone)- PCL (PES-PCL)	rhBMP-2	Results from chondrogenic differentiation of cells on scaffolds indicated that the lower modulus PCL fibers provided more appropriate microenvironments for chondrogenesis, by upregulation of Sox9, collagen type II and aggrecan gene expression and sGAG production compared to core-shell PES-PCL fibers	(97)
**Human**	Bone Marrow	MSCs	PLLA	TGF-B1, IGF-1, Dexamethasone, Ascorbic acid-2-phosphate, Sodium Pyruvate, L-proline, ITS+1 premix	The mRNA levels of aggrecan and collagen type II in TGF-B1/IGF-I treated cultures were notably higher than those treated only with TGF-B1, although these differences were not statistically significant. However, collagen type II/collagen type I ratio was high in TGFB1/IGF-I treated cultures. Also, in tow conditions, both sGAG and hydroxyproline accumulation showed significant changes over culture time	(98)
**Human**	Bone Marrow	MSCs	PCL nanofibers	TGF-B1, Ascorbic acid-2-phosphate, Dexamethasone, Sodium Pyruvate, ITS+1 premix	Constructs cultured in the presence of chondrogenic medium supplemented with TGF-B1 revealed significantly upregulated expression of aggrecan and Collagen type II and also abundant proteoglycan-rich ECM compared to constructs cultured in the presence of chondrogenic medium alone	(99)
**Human**	Articular Cartilage	Chondrocytes	Micro and Nanofibers PLLA	TGFB1, ITS+1 premix, Dexamethasone, Ascorbic acid-2-phosphate	In both types, scaffolds indicated an increase in sGAG production and Collagen type II expression over time	(100)
**Canine**	Articular Cartilage	Chondrocytes	Electrospun poly(D,L-lactide)/poly(L lactide) (PDLA/PLLA) or poly(D,L lactide)/polycaprolacton e (PDLA/PCL) with chitosan-based hydrogel	Ascorbic acid-2-phosphate	Primary canine chondrocytes produced collagen type II and proteoglycans while being cultured on scaffolds composed of electrospun PDLA/PCL and chitosan hydrogel	(101)
**Rabbit**	Articular Cartilage	Chondrocytes	PLLA nanofibers modified with cationized gelatin (CG) (CG-PLLA)	-	*In vitro* studies indicated that CG-PLLA could enhance viability, proliferation and differentiation of rabbit articular Chondrocytes compared with pristine PLLA nanofibers. In addition, these cell–scaffold constructs were able to maintain the expression of characteristic markers (collagen II, aggregan and SOX 9) of chondrocytes	(102)
**Human**	Bone Marrow	MSCs	PLLA nanofibers	TGF-B1, Ascorbic acid-2-phosphate, L-proline, Dexamethasone, Sodium Pyruvate, ITS+1 premix	PLLA-scaffold seeded with MSCs transfected with SOX-9 showed an increase in aggrecan mRNA expression over controls	(103)
**Human**	Bone Marrow	MSCs	PLGA nanofibers	Chondrogenic induction medium (CM, hMSC Differentiation BulletKit-chondrogenic, Lonza), TGF-B3	Production of proteoglycan and type-II collagen and also the high expression levels of SOX9 and COL10A1 were observed in differentiated BMMSCs on nanofibers in comparison to two-dimensionally cultured cells	(61)
**-**	-	ATDC5 chondrogenic cell line	Collagen-PLA, Collagen-PLGA	-	The addition of collagen has a dual influence of making the scaffolds more hydrophilic and reinforcing the mechanical properties. Furthermore, the soft scaffolds composed of the highly biodegradable PLGA50:50 and collagen, in two ratios (40:60 and 60:40), were optimal for chondrogenesis with ECM production and enhanced cartilage specific gene expression	(62)
**Human**	Articular Cartilage	Chondrocytes	poly(3hydroxybutyrate)/poly (3hydroxyoctanoate) P(3HB)/P(3HO)	-	The finding revealed that two ratios of P(3HB)/P(3HO) enhanced the aggregation of hyaline-like cartilage matrix and type II collagen after three weeks of culture with chondrocytes	(63)
**Rabbit**	Articular Cartilage	Chondrocytes	PLLA/ silk fibroin (PLLA/SF)	-	The PLLA/SF composite scaffold supports adhesion, proliferation, and growth of chondrocyte higher than PLLA scaffold without SF	(64)

**Figure 2.**
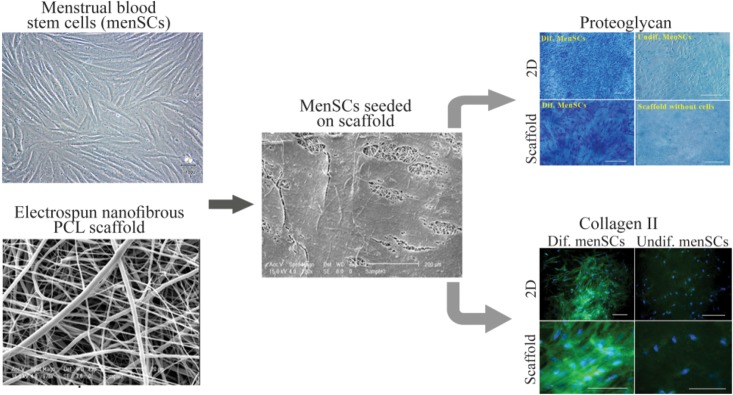
Culture and chondrogenic differentiation of MenSCs on nanofibrous scaffold. The image analyses of the scanning electron microscopy show that cells penetrated and adhered well on the surface of the mesh. Development of cartilage-like tissue in cultured constructs has been examined histologically with respect to the presence of proteoglycan and collagen type II (Scale bar: 100 *μm*). PCL: Polycaprolactone, Dif: Differentiated, 2D: Two Dimensional. (Adopted from Kazemnejad *et al* 2014 ^40^, with minor modification).

Our group has demonstrated that MenSCs, with higher proliferation capacity than BMMSCs, have the potential to undergo chondrogenic differentiation on PCL nanofibers ^56,57^. In addition, culturing on PCL nanofibers improved level of sGAG and proteoglycan production compared to PCL film (Figure 2).

Dahl *et al* investigated the potential of human Umbilical Cord Mesenchymal Stem Cells (UCMSCs) for chondrogenic differentiation on PLGA and PCL electrospun nanofibers ^48^. Cell culturing on nanofibers resulted in the production of higher levels of sGAG and sulfated proteoglycans. The ratio of collagen type II to type I expression was considered as the differentiation index (DI) in cartilage tissue engineering. There was a significant increase in the DI between PLGA and pellet control while no differences between PCL and PLGA cultures or between the PCL and pellet cultures were detected. While the expression level of elastin was not different between pellet controls and the two nanofiber conditions, significant increase in collagen type X on PLGA nanofiber scaffolds was found when compared to pellet controls.

### Hybrid nanofibrous matrices

***Randomly-oriented nanofibers:*** Shafiee*et al* have proved the potential of hybrid PVA/PCL nanofiber mesh seeded with rabbit BMMSCs in terms of cartilage tissue engineering *in vitro* and *in vivo* ^3^. Electrospinning PVA concurrently with PCL improved the capacity of nanofibrous scaffold for cell attachment and interactions and consequently improved cell proliferation rate.

In another study, Ahmed *et al* suggested that soft scaffolds composed of the highly biodegradable PLGA and collagen, in two ratios (40:60 and 60:40) were optimal for chondrogenesis ^62^. Most recently, Ching *et al* suggested that P(3HB)/P(3HO) nanofiber scaffolds fabricated by electrospinning reduce the risk of developing secondary osteoarthritis and may be suitable for clinical use ^63^. Moreover, it has been newly indicated that the PLLA/silk fibroin (PLLA/SF) composite scaffold supports adhesion, proliferation, and growth of chondrocyte more than PLLA scaffold without SF, introducing this scaffold a suitable material with potential application in cartilage tissue engineering ^64^.

***Aligned nanofibers:*** A study has been conducted on seeding BMMSCs and fibrochondrocytes on PCL-PEO aligned nanofibrous meshes and demonstrated that aligned nanofibrous topography could influence human BMMSCs fibrochondrogenesis by mimicking the naturally-occurring ECM more closely than micro-patterned features such as ridges or grooves ^65^.

In this way, Shafiee *et al* evaluated cell proliferation and chondrogenesis on aligned (A) and randomly (R) oriented electrospun PLLA/PCL hybrid scaffolds. They demonstrated that NSPs exhibit different behavior in two scaffolds. NSPs seeded on R fibers were expanded in all directions and exhibited a polygonal morphology and displayed multipolar shape. Conversely, NSPs were oriented only in the longitudinal direction of A fibers and showed bipolar extension along the fiber course alignment. Also, NSPs cultured on A fibers showed significantly higher expression of markers related to chondrogenesis process compared to cells cultivated on R fibers. The authors emphasized the role of the physical and topographical characteristics of scaffolds in the development of efficient stem cell-scaffold complexes and concluded that the aligned nanofibrous scaffolds can significantly enhance chondrogenic differentiation of nasal septum derived progenitors ^66^.

### Micro-nano fibrous scaffolds

While nanoscale features are desired due to mimicking the ECM components such as collagen fibers, it is believed that high concentrations of nanoscale fibers could increase cell spreading and limit cellular infiltration ^67,68^. Therefore, fabrication of multi-scale scaffolds combining microfibers with nanofibers has been considered with the aim of providing larger pore sizes and improving cell differentiation and ECM production ^69^. For this purpose, Levorson *et al* fabricated electrospun scaffolds consisting of two differently scaled fibers interspersed evenly throughout an entire construct as well as scaffolds containing fibers of fibrin and PCL. The prepared samples were scaffolds containing PCL microfibers (Pμ), PCL microfibers with PCL nanofibers (PμPn), and PCL microfibers and fibrin nanofibers (PμFn) being electrospun by a dual extrusion process. Both PμFn and PμPn scaffolds displayed similar porosities higher than microfibers alone. However, the Pμ scaffolds bore significantly larger pore sizes than the scaffolds containing nanofibers. Additionally, the PμPn scaffolds had the highest density of nanofibers and the smallest average pore size of all samples. The seeded human UCMSCs on PμPn scaffolds appeared to exhibit a flattened, broad polygonal morphology and spread along microfibers while cells on the Pμ and PμFn scaffolds showed more elongated and spindle-like morphologies and extended between the microfibers. Furthermore, analysis of cellular infiltration by Fast Green staining showed more scattered distribution of cells within the PμPn scaffolds while cells were primarily located on the surface of the other two scaffold types. Histological examination also exhibited more deposition of sGAG in PμPn and PμFn in contrast to the scaffolds composed of microfibers alone suggesting that the inclusion of nanofibers within a microfiber is useful towards the production and distribution of sGAG and may be beneficial for cartilage regeneration. The authors emphasized on tuning the density of nanofibers with respect to microfibers in an effort to control the positive influence of nanofibers on cell attachment and ECM production while minimizing any negative effects such as limited infiltration ^70^.

### Three dimensional nanofibrous scaffolds

To achieve 3D highly porous nanofibrous structure for cartilage tissue engineering, Hu *et al* used a phase separation method to fabricate a desirable scaffold made of PLLA. They showed that fabricated nanofiber scaffolds could efficiently support chondrogenesis of human BMMSCs in the presence of TGF-β1. The expression of chondrogenic markers in human BMMSCs grown on nanofiber matrix was significantly higher compared with cells raised on smooth film culture ^13^.

Li *et al* examined the differentiation of adult BMMSCs to chondrocytic phenotype on a nanofibrous PCL scaffold. They found that in Nanofibrous Scaffold (NFS) chondrocyte-like cells produced higher level of cartilaginous ECM compared with high-density Cell Pellet (CP) culture. In addition, specifically, collagen type IX was expressed to an upper level in nanofibrous system compared to CP culture. Furthermore, the level of sulfated Glycosaminoglycan (sGAG) synthesis in NFS culture was over two-fold higher than CP culture over a 21-day culture period. Their experimental results suggested that, while a 3D environment and TGF-β1 were both necessary to induce chondrogenesis, the PCL-based NFS significantly enhanced the chondrogenic differentiation of BMMSCs compared to the CP culture and could be considered as a candidate scaffold for cell-based tissue engineering approaches to cartilage repair compared to the CP culture ^55^.

### Biomolecules-loaded nanofibrous structures

The potential of electrospun nanofibrous and micro-fibrous PCL scaffolds to release TGF-β1 and stimulate chondrogenic differentiation of BMMSCs has also been investigated by Schagemann *et al*. They found that the augmentation of nanofibrous texture with or without TGF-β1 and/or hyaluronan was helpful in terms of directing the implanted BMMSCs toward a chondrogenic end. In addition, their results demonstrated that nanofibrous scaffold groups have different trends with microfibrous scaffolds via release level of TGF-β1 and chondrogenic development. The microfibrous scaffolds release TGF-β1 more than nanofibrous scaffolds; however, expression of cartilage marker in nanofibrous scaffold groups was higher than that in microfibrous scaffolds ^48^.

Recently, injectable microspheres were suggested as an attractive stem cell and growth factors carriers for tissue regeneration. In a study by Zhang *et al*, Transforming Growth Factor-β1 mimicking peptide cytomodulin (CM), was conjugated onto the functional nanofibrous hollow microspheres (FNF-HMS) to induce distinct differentiation pathways of rabbit BMMSCs. Their finding indicated that novel FNF-HMS effectively presents CM to BMMSCs and successfully induces their chondrogenesis for cartilage formation in both *in vitro* and *in vivo* studies ^71^.

## *In vivo* studies on repair of cartilage defects using constructs composed of nanofibers and stem cells

### Single polymer-based nanofibrous matrices

Implantation of nanofibers-based tissue engineered cartilage eliminates the need for an extra covering material to secure and protect the implant, such as periosteum which is used in the current autologous chondrocyte transplantation procedure. Harvesting periosteum comes with morbidity and complications, thus it is clinically preferable to avoid the use of periosteum ^72^.

In the recent decade, effectiveness of implanted electrospun PCL nanofibrous scaffold with/without cells has been evaluated for repair of cartilage defects in animal models (Table 3). Li *et al* demonstrated the potential of BMMSCs-seeded PCL-based nanofibrous scaffolds to repair full-thickness cartilage defects in a swine model. This cell-scaffold construct renewed hyaline cartilage-like tissue and restored a smooth cartilage surface as compared with other groups, including acellular constructs and untreated group. Furthermore, the studied group, which was chondrocyte-seeded scaffold, produced fibrocartilage-like tissue with an irregular superficial cartilage contour ^72^.

**Table 3.** *In vivo* studies for repair of cartilage defects using constructs composed of nanofibers and stem cells

**Host**	**Cells source**	**Cells type**	**Biomaterials**	**Stimulating factors**	**Results**	**Ref.**
**Pig**	Bone Marrow	Mesenchymal Stem Cells (MSCs)	Hyaluronate/type I collagen/fibrin composite Scaffold containing polyvinyl alcohol (PVA) nanofibers and	FGF-2 and Insulin	The cell-free composite scaffold improved migration of the bone marrow stem cells into the defect, and their differentiation into chondrocytes and also enhanced the regeneration of osteochondral defects towards hyaline cartilage and/or fibrocartilage in contrast to control cases that were left untreated and were filled with fibrous tissue	(1)
**Rabbit**	Bone Marrow	MSCs	Collagen and Polyl-Lactic Acid (PLA)	-	Compared with collagen scaffold, implantation of collagen-nanofiber scaffold seeded with cells induced more rapid subchondral bone appearance, and better cartilage development, which led to better functional repair of deep osteochondral defects in rabbits	(2)
**Rabbit**	Bone Marrow	MSCs	PVA/ poly (ε-caprolactone) (PCL) nanofiber (PVA/PCL)	-	A high similarity in ECM patterns between regenerated tissue in the group which received cell-seeded scaffold and normal tissues was observed. Also, the production of collagen type II in these groups was high compared to other groups	(3)
**Rat**	-	-	Poly (Vinyl Alcohol) - methacrylate (PVA-MA) and Chondroitin Sulfate (CS)	-	CS fibers combined with PVA fibers induced statistically higher type II collagen production compared with the PVA fibers alone and empty defects	(7)
**Swine**	Articular Cartilage	Allogeneic Chondrocytes	PCL	-	In contrast to acellular constructs and the no-implant control groups, MSC-seeded scaffolds renewed hyaline cartilage-like tissue and restored a smooth cartilage surface. In addition, the chondrocyte-seeded scaffolds produced fibrocartilage-like tissue with an irregular superficial cartilage contour	(72)
**Human**	Bone marrow	Xenogeneic MSC				
**Rabbit**	-	-	PCL with Chitosan	TGF-B1, Ascorbate-2-phosphate	Cartilage formation and production of sGAG in the uncoated scaffolds increased at the end of implantation time compared to chitosan-coated scaffolds. Also, significantly more mineral dep osition was detected inTGF-β1-injected and uncoated scaffolds compared to vehicle-injected and coated scaffolds	(73)
**Rabbit**	Bone Marrow	MSCs	oriented poly (L-lactic acid)-copoly (e-caprolactone) P(LLA-CL) yarn collagenI/hyaluronate hybrid scaffold (Yarn-CH) as a chondral phase and Porous beta-TCP as a osseous phase	TGF-B1, Dexamethasone, Ascorbate-2-phosphate,L-proline, Sodium pyruvate, ITS+1 Premix	In differentiated MSCs/YarnCH/TCP and MSCs/CH/TCP biphasic scaffold groups, the regenerated defects almost completely full with hyaline-like repaired tissue appeared to be integrated with the surrounding tissues. In undifferentiated MSCs/YarnCH/TCP and MSCs/CH/TCP biphasic scaffold groups, defects were covered by rough tissue with irregular surfaces which were clearly distinguishable from the normal cartilage. Furthermore, immunohistochemical staining showed high level of collagen type II in the BMSCs/YarnCH/TCP biphasic scaffold groups than in the other groups	(76)
**Rabbit**	-	-	porous hydroxyapatite/collagen (HAp/Col) scaffold	FGF-2	Abundant bone formation was observed in the HAp/Col implanted groups as compared to the control group. Furthermore, HAp/Col impregnated with FGF-2 displayed not only abundant bone regeneration but also the most satisfactory cartilage regeneration, with cartilage presenting a hyaline-like appearance	(78)

### Hybrid nanofibrous matrices

An autologous cell-based cartilage repair approach has been developed to eliminate harvesting of healthy cartilage and *in vitro* culture. In this study, PCL nanofiber scaffolds ^73^ (with/without chitosan coating) were implanted under periosteum in six months old rabbits with injection of GF-β1 into the implant site. Cell infiltration was observed in all groups while sGAG production and cartilage formation was more typical in the uncoated scaffolds compared to chitosan-coated scaffolds. In addition, TGF-β1-injection and application of uncoated scaffolds resulted in significantly more mineral deposition.

The iPSCs can be produced by reprogramming of terminally differentiated cells to primary stem cells with pluripotency. To benefit from the breakthrough of iPSCs, the effect of electrospun PCL/gelatin nanofibrous scaffolds on the chondrogenesis of iPSCs and articular cartilage defect restoration was investigated. It was indicated that iPSCs expressed higher levels of chondrogenic markers on the scaffolds than the culture plate. Additionally, in an animal model, cartilage defects implanted with the scaffold-iPSCs composite exhibited an enhanced gross appearance and histological improvements, higher cartilage-specific gene expression and protein levels, as well as subchondral bone regeneration. Therefore, it was shown that scaffolds enhanced the chondrogenesis of iPSCs and that iPSCs-containing scaffolds improved the re-establishment of cartilage defects to a greater degree than did scaffolds alone *in vivo* ^74^.

In another study, efficiency of the fabricated hybrid PVA/PCL nanofibers seeded with autologous BMMSCs was evaluated in the knees defect of rabbits. The authors indicated improved regeneration of cartilage in full-thickness defects that treated with BMMSCs-loaded PVA/PCL electrospun scaffolds compared to scaffold alone or untreated defects. After 12 weeks of implantation, almost all defects that were treated with cell-scaffold constructs were completely enclosed with smooth tissue and edges of the grafted areas were hardly detectable. In addition, unlike the group who received only PVA/PCL scaffolds, a high similarity in ECM patterns between regenerated and normal tissues was observed and collagen type II staining was positive ^3^.

In another study by He *et al*, the cell seeded electrospun nanofibers containing collagen-poly (L-lactic acid-co-ε-caprolactone) (collagen-PLCL) and chondrocytes were implanted subcutaneously into nude mice followed by evaluation of the quality of neocartilage. Their results revealed that collagen-PLCL membranes facilitate the formation of cartilage-like tissue in animals and thus could mimic the natural ECM with good cell affinity ^75^.

Recently, bi-layer scaffolds have gained considerable attention for the restoration of osteochondral defects affecting both the articular cartilage and the underlying subchondral bone ^74^. Combination of collagen and electrospun nanofibers as bi-layer scaffold has been demonstrated to help cartilage and bone regeneration. In 2013, Zhang *et al* reported efficiency of a fabricated bi-layer microporous scaffold with collagen and electrospun PLLA nanofibers (collagen-PLLA) for repair of osteochondral defects. Compared with collagen scaffold, implantation of collagen-PLLA scaffold seeded with BMMSCs induced more rapid subchondral bone appearance and better cartilage development, which led to better functional repair of deep osteochondral interfacial tissue structure in rabbits ^2^.

Liu *et al* have also studied the efficiency of a biphasic complex to repair the osteochondral defects in a rabbit model, which was composed of oriented electrospun poly (L-lactic acid)-co poly(e-caprolactone) P(LLA-CL) Yarn-collagen type I/hyaluronate hybrid scaffold (Yarn-CH) as a chondral phase and porous beta tricalcium phosphate (TCP) as a osseous phase. The regenerated defects treated by differentiated BMMSCs/Yarn-CH/TCP and BMMSCs/CH/TCP (control) biphasic scaffold groups were completely repaired by hyaline-like tissue that appeared to be integrated with the surrounding tissues. In undifferentiated BMMSCs/Yarn-CH/TCP and BMMSCs/CH/TCP biphasic scaffold groups, defects were covered by rough tissue with irregular surfaces which were clearly distinguishable from the normal cartilage. In addition, the cell distribution and cell morphology in the regenerated cartilage were almost identical to the native host cartilage including the superficial zone in differentiated groups compared to undifferentiated groups. Furthermore, immuno-histochemical staining showed higher level of collagen type II in the BMMSCs/Yarn-CH/TCP biphasic scaffold groups than in the other groups. Greater improvement of the compressive modulus was also shown in differentiated-BMMSCs/Yarn-CH/TCP biphasic scaffold group compared to other groups ^76^.

### Three dimensional nanofibrous scaffolds

In 2010, a three-dimensional PLGA/nano-hydroxyapatite (PLGA/NHA) scaffold was fabricated by a thermally induced phase separation method and its efficacy to repair articular osteochondral defects in murine model was investigated. The defects in the PLGA/NHA-MSCs treated group were filled with smooth and hyaline-like cartilage with profuse glycosaminoglycan and collagen type II deposition12 weeks after operation ^77^.

For interacting cells with the surrounding ECM, which gives rise to a dynamic transfer of information between the extracellular and intracellular space, researchers have introduced several biological signals, including chondroitin sulfate, hyaluronic acid, and collagen into tissue-engineered scaffolds to encourage tissue specificity ^43^. In 2012, 3D electrospun nanofiber network composed of PVA-methacrylate (PVA-MA) and a composite of PVA-MA/chondroitin sulfate-methacrylate (CS-MA) (PVA-MA/CS-MA) were used for articular cartilage repair. After evaluation of the scaffolds for cartilage-like tissue formation *in vitro*, the constructs were implanted into rat osteochondral defects. Their results showed that nanoscaffolds with chondroitin sulfate (PVA-MA/CS-MA) supported chondrogenesis more than PVA-MA alone, judged by collagen type II and proteoglycan production in defects ^43^.

### Biomolecules-loaded nanofibrous structures

Some investigators evaluated impregnation of nanofibrous scaffolds with various growth factors to promote repair of articular cartilage defects. For example, Maehara *et al* assessed efficacy of a bi-layer porous hydroxyapatite/collagen (HAp/collagen) nano-composite prepared by coprecipitation method and impregnated with FGF-2 for repairing the osteochondral defects in a rabbit model. Their results showed that HAp/collagen scaffolds impregnated with FGF-2 not only regenerate bone tissue but also resulted in development of satisfactory cartilage regeneration with a hyaline-like appearance. Their finding suggested that porous HAp/collagen with FGF-2 augmented the cartilage repair ^78^ .

Filova *et al* developed a novel drug delivery system using nano-composite for repair of osteochondral defects in miniature pigs. The fabricated cell-free composite scaffolds that contained PVA nanofibers enriched with liposomes, FGF-2, and insulin were subsequently embedded in a fibrin gel including hyaluronate/collagen type I. Interestingly, the cell-free composite scaffold improved migration of the BMMSCs into the defect and their differentiation into chondrocytes as compared with untreated group. As a result, composite scaffold containing nanofibers with liposomes functionalized with growth factors was able to enhance the regeneration of osteochondral defects towards hyaline cartilage and/or fibrocartilage compared with untreated defects that were filled predominantly with fibrous tissue ^1^ .

## Conclusion

One key challenge in tissue engineering especially cartilage reconstruction is mimicking the architecture of ECM. At present, nanofibrous scaffolds irrespective of their method of synthesis are the most promising matrix to generate artificial ECM. These scaffolds are characterized by high surface area and enhanced porosity, which are highly desired for tissue engineering and drug delivery applications. There are four dominant methods to fabricate nanofibers for cartilage tissue engineering: electrospinning, molecular self-assembly, phase separation, and drawing ^8,14^.

Of these methodologies, electrospinning is the most common approach for cartilage tissue engineering since this technique offers great flexibility in terms of the choice of scaffold material and fiber diameter from the micrometer down to nanometer range. Electrospun polymeric fibrous meshes also present a higher surface area for cell attachment. Indeed, fabrication of electrospun nanofibers is easy, inexpensive and relatively reproducible ^79,80^. Due to difficulties in controlling porosity and pore size and architecture, three other techniques have been utilized less than electrospinning for cartilage tissue engineering purposes.

The availability of a wide range of natural and synthetic biomaterials has broadened the scope for development of nanofibrous scaffolds. Synthetic polymer-based systems offer additional advantages with their adjustable mechanical properties, as well as ease of surface modification via protein coatings, or conjugation of specific signaling molecules ^81^. The most common electrospun nanofibers designed for cartilage tissue engineering are made of poly (α-hydroxyesters) ^82^. Although the synthetic nanofibers prepared form these materials are capable to support chondrocyte proliferation and differentiation, some strategies have been applied to improve cell tendency of these materials that help us to achieve better results in future repair of cartilage defects. One applied strategy is hybridization of these synthetic materials by natural polymers like collagen ^62^. The combination of synthetic materials with natural polymers in nanofibers has resulted in better cell attachment, proliferation and chondrogenic development compared to synthetic polymers alone ^64^. Moreover, some *in vivo* studies implied better repair of cartilage defects by hybrid nanofibers compared to simple nanofiber composed of only synthetic polymers. Another strategy is modifying the surface of scaffolds through physical and chemical methods to improve the bioactivity of materials for cell adhesion and distribution.

**Figure 3:**
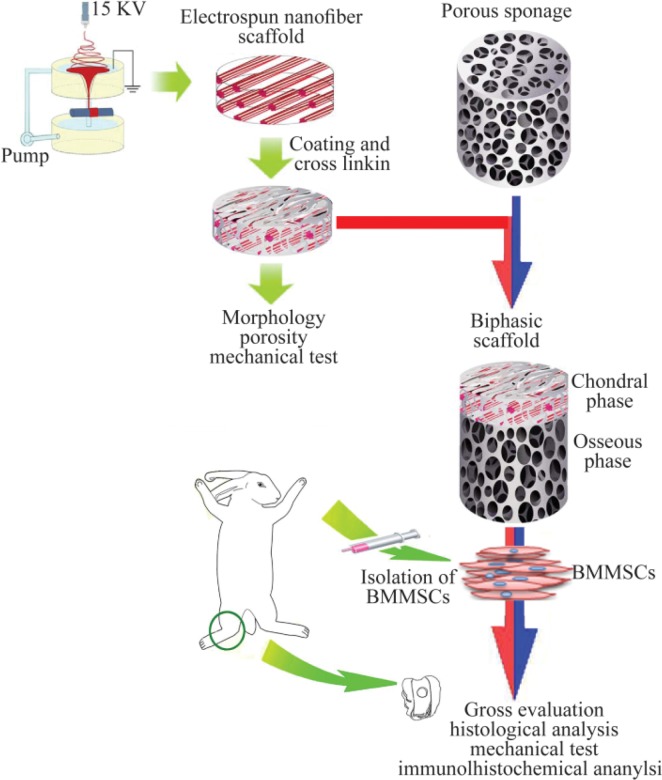
A schematic model for *in vivo* study on repair of osteochondral defects using constructs composed of nanofibers and stem cells. The nanofiber is considered as the chondral phase. Porous sponage is used as the osseous phase. After combining with BMMSCs, biphasic complex was utilized to repair osteochondral defects in the animal model (Adopted from Liu *et al* 2014^56^, with modification).

One approach to improve cell affinity is surface modification of the nanofibers by plasma treatment ^83^. Some others have improved the cell affinity of nanofibers by attaching Arg-Gly-Asp (RGD) peptides to the nanofibers surfaces. Indeed, these strategies play an important role in governing cellular responses and helping the scaffold to play a more efficient role as bioactive systems rather than just passive cell carriers. To prove this assumption, different *in vitro* and *in vivo* studies narrated that integration of fabrication techniques with surface modification methods has resulted in closer properties of nanofibrous scaffolds to native ECM, encouraged cell attachment and development into chondrocyte lineage. To repair the osteochondral defects that have two different structures, incorporation of stem cells with biphasic scaffolds containing hybrid nanofibers for chondral phase and porous sponge scaffolds for osseous phase seems to be a good strategy (Figure 3).

In spite of the great achievements behind the design of nanofibrous scaffolds, there is still plenty of room for improvement. Integration of nanofibers into micro-fabricated 3D scaffolds has resulted in obtaining more desirable scaffolds with providing larger pore sizes and improving cell differentiation and ECM production.

The future research on nanofibrous architecture may be focused on the new nanofabrication techniques. In combination with new nanofabrication technologies, nanofibrous scaffold could be decorated with nano-topographic patterns, such as ridges and grooves to better match the nanostructure of ECM achieving a better control of ECM-mimicry.

Based on this review, the efficiency of cell-seeded nanofibers in repair of cartilage defects is significantly more than the scaffold alone. It sounds that the seeded cells *via* secretion of growth factors and cytokines help sGAG production and mediate better situation to mimic ECM environment ^8^.

Two primarily considered criteria to determine the optimal source of cells for cartilage repair are the performance of the cells and their accessibility. Regarding performance, primary or low passage articular chondrocytes provide several advantages due to their high level of matrix synthesis and lack of hypertrophy. However, for larger defects, which require a larger number of cells, it is generally accepted that the dedifferentiation which occurs during monolayer expansion is a significant hurdle ^84^.

On the other hand, due to requirements of two-step intra-articular procedures for clinical use of autologous chondrocytes, one to harvest the cartilage and one to re-implant, many groups are attempting to develop allogenic sources of cells to be used in articular cartilage repair. Although cartilage is considered an immune privileged site, newer data indicate that chondrocytes have immunological properties that limit host immune reaction ^85^.

Following the search for immune privilege cell source that can readily provide large numbers of undifferentiated progenitors with chondrogenic potential, adult stem cells were introduced as interesting cells for tissue engineering and regenerative medicine purposes.

The most commonly used stem cells for cartilage tissue engineering especially in nanofibrous structures are the stem cells derived from bone marrow. It is due to the high chondrogenic differentiation ability and the availability of great knowledge about immunological characteristics and nature of this source of stem cells compared to other adult mesenchymal stem cells ^5,86^. However, due to some problems such as invasive techniques for sample collection and low availability, BMMSCs are introduced as not an ideal source and still some challenges for tissue engineering application exist. With introducing more available and accessible stem cell sources with similar immunological properties and great proliferation and trans-differentiation ability such as menstrual blood and adipose tissue stem cells, it is expected that these newer stem cells would be synchronized with nanofibers for future studies on cartilage tissue engineering.

Notably, besides improvement of nanofiber fabrication technique, utilization of other stem cell sources instead of BMMSCs and incorporation of nanofibers with differentiation promoting growth factors such as BMP-6 ^87^ are future research priorities of cartilage reconstruction. In this manner, designing and applying suitable bioreactors that ultimately help in more ECM production and achievement of artificial constructs simulating native cartilage tissues, should not be ignored. In conclusion, although many experiments have been carried out to simulate native cartilage using nanofibers and stem cells with some promising reports about efficiency of these constructs for repair of cartilage defects in animal models, much joint effort by scientists from multiple disciplines is still required for transition of the data from *in vitro* to *in vivo* phase. To facilitate the future applicability of constructs composed of stem cells and nanofibers, a time frame is required for development of bench to bedside strategies.
